# Neuroligins Nlg2 and Nlg4 Affect Social Behavior in *Drosophila melanogaster*

**DOI:** 10.3389/fpsyt.2017.00113

**Published:** 2017-07-10

**Authors:** Kristina Corthals, Alina Sophia Heukamp, Robert Kossen, Isabel Großhennig, Nina Hahn, Heribert Gras, Martin C. Göpfert, Ralf Heinrich, Bart R. H. Geurten

**Affiliations:** ^1^Department of Cellular Neurobiology, Institute for Zoology and Anthropology, University of Göttingen, Göttingen, Germany

**Keywords:** *Drosophila melanogaster*, social behavior, activity monitoring, interindividual distance, sensory–motor functions, mutations, human neuro-developmental diseases

## Abstract

The genome of *Drosophila melanogaster* includes homologs to approximately one-third of the currently known human disease genes. Flies and humans share many biological processes, including the principles of information processing by excitable neurons, synaptic transmission, and the chemical signals involved in intercellular communication. Studies on the molecular and behavioral impact of genetic risk factors of human neuro-developmental disorders [autism spectrum disorders (ASDs), schizophrenia, attention deficit hyperactivity disorders, and Tourette syndrome] increasingly use the well-studied social behavior of *D. melanogaster*, an organism that is amenable to a large variety of genetic manipulations. Neuroligins (Nlgs) are a family of phylogenetically conserved postsynaptic adhesion molecules present (among others) in nematodes, insects, and mammals. Impaired function of Nlgs (particularly of Nlg 3 and 4) has been associated with ASDs in humans and impaired social and communication behavior in mice. Making use of a set of behavioral and social assays, we, here, analyzed the impact of two *Drosophila* Nlgs, Dnlg2 and Dnlg4, which are differentially expressed at excitatory and inhibitory central nervous synapses, respectively. Both Nlgs seem to be associated with diurnal activity and social behavior. Even though deficiencies in Dnlg2 and Dnlg4 appeared to have no effects on sensory or motor systems, they differentially impacted on social interactions, suggesting that social behavior is distinctly regulated by these Nlgs.

## Introduction

Molecular mechanisms that regulate cellular metabolism, development, differentiation, and survival are essentially shared by most animal species. Recent evidence suggests that the last common ancestor of vertebrates and invertebrates, the so-called urbilaterian, already possessed a centralized nervous system that contained the precursors of brain structures and neurosecretory organs of extant protostomes and deuterostomes (1, 2). In this respect, homologous structures have been identified between insect and mammalian brains, such as the mushroom bodies and the pallium or cortex (2), the central complex and the basal ganglia (3), the pars intercerebralis/pars lateralis/corpora cardiaca system and the hypothalamus–pituitary axis (4, 5), the corpora allata, and the adenohypophysis (anterior pituitary) (6). Moreover, genes implicated in human disease and the generation of social behaviors have well-conserved homologs in insects and other invertebrates (7–9).

Studies on the fruit fly, *Drosophila melanogaster*, have successfully contributed to the characterization of molecular pathways underlying human nervous system diseases, such as Rett syndrome, Parkinson’s disease, Alzheimer’s disease, and others (10–13). More recently, *Drosophila* has even been used to study the mechanistic basis of neuro-developmental diseases, such as fragile X syndrome (14, 15), autism spectrum disorders (ASDs) (16), and schizophrenia (17–19).

*Drosophila* may serve as a suitable organism to study the basis of these diseases since it performs elaborate social interactions, such as courtship (20, 21) and aggression with the establishment of social dominance (22, 23), uses intraspecific acoustic communication (24), establishes long-term memory in classical and operant learning paradigms (25), and performs sensory-motor tasks with great precision (26). *Drosophila* offers molecular and genetic tools to identify the functions of individual genes and proteins, their interaction partners within cellular/molecular pathways [recent summary (27)], and their impact on physiology and behavioral performance. Homologs of disease-related genes can be mutated globally or in particular tissues or cell types, and transgenic flies may express human genes with or without characteristically disease-related mutations. Whether, and how, such genetic alterations impact *Drosophila* social behavior needs to be assessed in a quantifiable manner.

Neuroligins (Nlgs) are postsynaptic adhesion molecules that typically associate with presynaptic neurexins to form bidirectional signaling complexes required for the correct formation, maturation, and functional adjustment of chemical synaptic connections between neurons (28–31). Additional neurexin-independent synaptic functions have also been reported [reviewed by Reissner et al. (32)]. In mammalian nervous systems, different Nlgs are differentially expressed at different types of synapses [reviewed in Ref. (33–35)]. Nlg1 is predominantly expressed at excitatory glutamatergic synapses, fostering the accumulation of postsynaptic density proteins and ionotropic and metabotropic glutamate receptors (36). Nlg2 is selectively expressed at inhibitory synapses, where it associates with gephyrin and recruits GABA or glycine receptors (37). Nlg3 and Nlg4 appear at both excitatory and inhibitory synapses with preferences of Nlg3 for GABAergic and Nlg4 for glycinergic synapses (38–42). Alterations in *nlg* genes were found in patients affected by ASDs (40–43) and mutations of the same genes caused autism-like phenotypes in rodent model organisms (35, 44). ASD represent neuro-developmental disorders that cause impairments in social interaction and communication accompanied by restricted and repetitive behaviors. Especially mutations in *nlg3* and *nlg4*, mutations in genes encoding direct interaction partners of Nlgs, such as *neurexins* and *shank*, and alterations of other proteins involved in synaptic mechanisms are directly associated with ASD (44–46). Based on the differential expression of Nlgs at different types of synapses, it was hypothesized that ASD phenotypes may result from disturbed balance of excitatory and inhibitory synaptic transmission in brain regions controlling respective behaviors and functions (47). Supporting evidence for this hypothesis derived from both ASD patients [review by Dickinson et al. (48)] and studies on rodent models for ASD (46, 49–52).

Much like in mammals, insects present multiple *neuroligin* genes (38), whereby the four genes found in *D. melanogaster* (*nlg1*–*nlg4*) show differential expression at central and peripheral nervous synapses. None of these genes has a particular similarity to the four mammalian *nlg* genes and the designations (vertebrate *nlg1–4* vs *Drosophila nlg1–4*) do not imply phylogenetic relatedness. Previous studies have shown that Dnlg2 is predominantly expressed by excitatory postsynapses (53), while Dnlg4 is abundant at inhibitory synapses (54). Immunostaining with a Dnlg2 antibody in the adult *Drosophila* brain shows that the protein is abundant in the mushroom body and the central complex (W. Xie, personal communication). Both these brain structures are involved in the control of diverse behaviors like short-term courtship memory, center avoidance, olfactory learning, sleep regulation, and spatial orientation (55–59). Antibody staining against Dnlg4 revealed high expression in the lateral clock neurons (LNvs), possibly explaining the abnormal sleep behavior found in *dnlg4*-mutant flies, as well as expression in the central complex (54). Earlier studies (16) revealed that deletion of the *dnlg2* gene alters social behavior in *Drosophila*. While retaining intact sensory perception, Dnlg2-deficient flies display reduced social interactions with respect to male–female courtship and male–male agonistic behavior, produce altered acoustic communication signals, and often fail to terminate behavior upon context changes. In the present study, we subjected flies deficient in Dnlg2 (typically expressed at excitatory synapses) or Dnlg4 (typically expressed at inhibitory synapses) and wild-type *D. melanogaster* (wt) to a series of behavioral assays that assess their social interactions (courtship, aggression, group formation, reaction to conspecific songs) and, in addition, analyzed their acoustic communication signals. We also tested for motor and sensory defects by analyzing locomotion, open space avoidance, circadian activity, and the sound sensitivity or their hearing organs. Our results show that both Dnlg2 and Dnlg4 are implicated in *Drosophila* social behavior.

## Materials and Methods

### Animals

All flies were reared at 25°C temperature with 60% humidity under a 12:12-h dark/light cycle and on standard medium, which was made from 500 g fresh yeast, 500 g sugar, 20 g salt, 60 g agarose, 250 g flour, 1 l conventional apple juice (Alnatura, Bickenbach, Germany), and 30 ml propionic acid. Water was added so that the medium would amount to 7 l. Studies were performed with the Dnlg2-deficient mutant line *dnlg2*^KO17^ (provided by Wei Xie, Southeast University, Nanjing, China), generated by targeted knockout of the *dnlg2* genomic locus (53). Studies with a second Dnlg2-deficient line [*dnlg2^KO70^*; (53)] that was tested in some of the assays generated qualitatively similar results. The Dnlg4-deficient mutant line was generated by crossing a *dnlg4^del^* (54) deletion and a *dnlg4* point mutation (*dnlg4^LL01874^*) line (both provided by Junhai Han, Southeast University, Nanjing, China) (54), as both of them are homozygously lethal. We note that both fly lines are hypomorphs and still express limited amounts of the respective Nlg (less than 30% compared to wild type). Canton-S was used as wild-type control, and all mutant lines were kept as “Cantonized” lab stocks (dngl2-mutants were outcrossed for six generations in-house; dnlg4-mutants were obtained as outcrossed). Unless otherwise stated, flies were tested at the age of 5–7 days.

### qRTPCR

RNA was isolated from 50 fly heads per strain (*dnlg4^LL01874/Def^, dnlg2^KO17^*, Canton-S, *w^1118^*; resulting in a total of 200 heads) using the “ZR Tissue and Insect RNA MicroPrep” Kit (Zymo Research Europe GmbH, Freiburg, Germany; #R2030). 1 µg RNA per sample was reversely transcribed to cDNA *via* the “QuantiTect Rev. Transcription Kit” (Qiagen, Valencia, CA, USA, Cat No./ID: 205311) for primer sequences (10 pmol/μl), see Table S2 in Supplementary Material. Three repetitions were run for each strain, with 10 ng of the respective cDNA diluted in 4 µl of water. We added 5 µl of SYBR Green (iQ™ SYBR^®^ Green Supermix 2×, Bio-Rad Laboratories GmbH, Munich Germany; #1708880) and 1 µl H_2_O containing 0.1 µl of the forward and backward primers. The total 10 sample were sealed with transparent wrapper foil and centrifuged for 2 min at 2,000 rpm. RT-qPCR was performed on a Bio-Rad MyiQ Single color RT PCR Cycler (Bio-Rad Laboratories GmbH, Munich, Germany) employing the following program cycle: 3 min 95°C, 10 s 95°C, 30 s 60°C, and 30 s 72°C. The cycles were repeated 40 times. We used Rpl32 as a reference gene for cDNA concentration.

### Acquisition and Analysis of Locomotion Data

**Figure 1:**
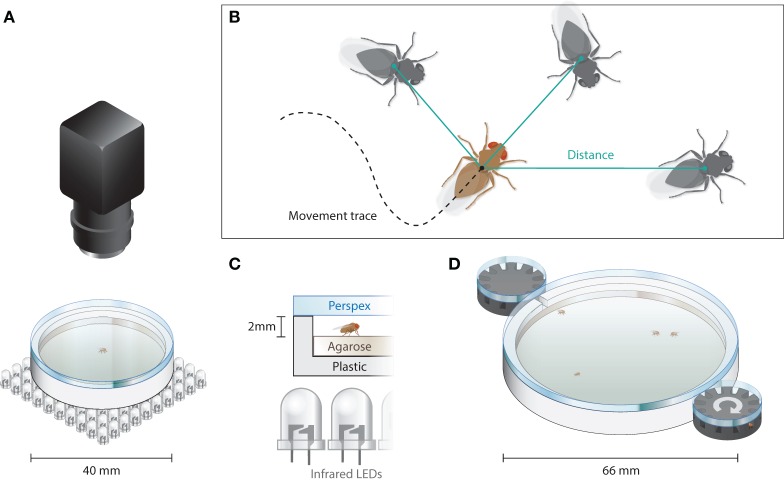
Data acquisition for locomotion and positional data. **(A)** Sketch of the walking arena used to assay locomotion and center avoidances [zoom-in in **(C)**]. Shadows of the flies were projected *via* a field of LEDs below the arena onto the camera. Visible light sources placed in the proximity **(B)**. Trajectories of individual flies were derived *via* the detection of ellipses (*Drosophila* body) in the difference image. Individual positions were used to determine interindividual distances between flies. **(C)** Close-up of the setup including the anti-glare Perspex pane, which was coated with Sigma-Aldrich Sigma Cote from the lower side, preventing the animal from crawling on the ceiling. **(D)** Social interaction arena, in which 24 flies were released one by one. The flies were released from the black revolvers, which are connected by a little tunnel to the main arena and hold 12 animals each. Every 90 s, one of the two revolvers would turn to release one fly.

Flies [*N*(*wt*) = 97, *N*(*dnlg2^KO17^*) = 96, *N*(*dnlg4^del/LL01874^*) = 66] were transferred individually in a circular arena of 40-mm diameter filled with 1% agarose/1% glucose and closed with an anti-glare Perspex plate. A distance of 2 mm between pane and medium allowed the flies to walk freely but prevented them from flying (see Figures 1A,C). The arena was produced using an Ultimaker 3D printer (Ultimaking Ltd., Geldermalsen, Netherlands) and movies were recorded using TroublePix software (NorPix Inc., Montreal, QC, Canada) and a MotionTraveller 500 camera (IS, Imaging Solutions GmbH, Eningen, Germany) at 500 frames per second (fps). The flies were illuminated from below with infrared LEDs (Pollin Electronic GmbH, Pförringen, Germany; #531090). Full LED illumination caused a temperature increase of ca. 0.01°C per minute. Because animals were allowed to spend maximally 5 min in the arena, the corresponding temperature change they experienced during their stay was ca. 0.05°C. *Post hoc* analysis of the video footage was performed using ivTools (Dr. Jens P. Lindemann; Bielefeld University) to acquire walking trajectories.

To deduce fly-based velocity combinations from the trajectory, we used unsupervised k-means clustering to classify data points into a set of “k” clusters (60). The fkmeans function for Matlab authored by Tim Benham was used.^1^

### Probability Density in a Circular Arena

Animals were set in the middle of a circular arena 40 mm in diameter (see Figure 1A and previous paragraph). Each animal was recorded for 10 s of consecutive walking to exclude effects caused by resting. To obtain probability densities, the Cartesian coordinates *x* and *y*, which were acquired through trajectory tracing, were transformed into polar coordinates with polar angle θ and radius *r*. We calculated the histogram of *r* for each fly and the median histogram for each strain, respectively. We then normalized that histogram for the surface area of each bin and normalized the resulting histogram so that its integral is 1, providing a probability density.

We also analyzed the median radius position by calculating the median *r* of each individual fly. The statistical difference between fly lines was calculated using a two-sided Kolmogorov–Smirnov test. *p*-Values were corrected *via* the Benjamini–Hochberg false discovery rate procedure (see Statistical Analysis).

### Circadian Rhythm

Circadian rhythm was analyzed using the *Drosophila* Activity Monitoring (Tritech Research, CircKinetics^2^) System. Flies were placed individually in glass tubes (diameter 3 mm; length 7 cm) and sealed with a gas permeable cap on one side and standard food on the other side. The food medium was identical to the rearing medium described before. The tubes were inserted into an incubator with a 12:12-h dark/light cycle, matching that of fly breeding incubator. Crossings of the midline were detected as interruptions of an infrared light beam and were automatically counted for 7 days. The first 48 h were omitted to avoid differences in behavior due to the relocation of the animal.

### Analysis of Social Distance and Group Behavior

Flies [*N*(*wt*) = 104, *N*(*dnlg2^KO17^*) = 94, *N*(*dnlg4^del/LL01874^*) = 119] were allowed to enter a circular aluminum walking arena of 66-mm diameter through two entrances on opposite sides (see Figure 1D). The arena was illuminated from below with LEDs (Nichia Cooperation, Tokushima, Japan; #NSSW157AT-H3). Each entrance was connected to a 12-chamber rotating revolver loaded with a single fly per chamber, allowing one fly at a time to enter the arena every 90 s. The positions of individual flies were determined at a frame rate of 50 fps, and the trajectories were analyzed afterward with ivTools (see Acquisition and Analysis of Locomotion Data).

To associate individual flies with a group, we used agglomerative hierarchical clustering. This algorithm uses the Euclidian distance between individual flies to determine their incorporation in a group. The agglomerative clustering runs showed a clear threshold at about 20 mm interindividual distance and, accordingly, animals being more than 20 mm away from the next fly were counted as not being part of a group. At this distance, a *Drosophila* fly extends the visual field of only one ommatidium (61). Only flies that gathered together for more than 30 s were counted as groups.

Male–male courtship and the formation of chains of multiple males following each other were scored from the videos by eye. The latter chaining behavior was only taken into consideration when a male followed another one with its wing extended for more than 3 s (most chains were stable for several minutes). The leading animal was not considered to be actively chaining and was, therefore, excluded. An example of chaining behavior can be seen in Figure S1A in Supplementary Material.

### Peripheral Auditory Functions

To test for possible defects in hearing, we affixed the flies with wax on a focus holder (62) and then measured vibrations of their antennal sound receiver (63).

Vibrations were measured at the tip of the antennal arista using a PSV-400 Laser-Doppler-Vibrometer (Polytec GmbH, Waldbronn, Germany). For acoustic stimulation, pure tones were broadcasted *via* a loudspeaker positioned ca. 10 cm behind the fly. The stimulus frequency was adjusted to match the individual best frequency of the fly’s receiver as determined from the power spectrum of its vibrations in the absence of sound (64). Electrophysiological recordings of compound action potentials of auditory receptor neurons were performed with an etched tungsten electrode inserted between antenna and head (65).

### Sound Recordings

Male courtship songs (CSs) in the presence of a decapitated, 5- to 7-day-old virgin female were recorded using a microphone (Bruel & Kjaer Type 4165) in a soundproof chamber. The recorded signals were amplified, band bass filtered (70–5,000 Hz), and directly digitized with a sampling frequency of 44,100 Hz. For acquisition, Audacity 2.0.6^3^ was used. Analysis was done using custom made MatLab programs. To determine the dominant frequency components of the songs, Fast Fourier Transformation using a 4096 Hanning window was applied.

### Competitive Courtship Assay with Acoustic Stimulation

Competitive courtship assays were performed with two socially naïve males (age 7–12 days) placed together with a decapitated, 5- to 7-day-old, virgin wt female in a circular arena with 2 cm diameter. The age disparity in male flies should have limited effects on the mating behavior, in this case. As the female is decapitated and mating is never successful, the attractiveness of males does not influence the female’s choice as described for different male age groups (66, 67). The initiation of courtship by the male does not vary much between the 7th and 14th day of age (68). The bottom of the arena consisted of a fine mesh. During the experiments, flies were exposed to either white noise (WN), aggression songs (AS), or CSs that were previously recorded from wild-type males (69). Acoustic stimuli were presented by a loudspeaker situated below the arena. Videos of the experiments were recorded for 15 min with a frame rate of 30 fps. Only the periods from 5 to 10 min after start of the experiments were considered.

Frame-by-frame analysis of recorded videos was performed by an observer unaware of stimulus conditions and fly strain. For each frame either idle, unilateral wing extension as a hallmark of courtship or aggression behavior was allocated to the acting individual fly. This was done using an in-house developed software tool that allows for fast video annotation *via* a game pad. This system allowed for long scoring sessions and high throughput *via* the observers, who scored 9,000 frames per replicate. Aggressive behavior directed against the other male was recognized by aggressive acts like boxing, leg kicking (see Figure 7), and production of agonistic sound signals with both wings elevated. Courtship behavior toward the female was identified by unilateral wing extension associated with the production of CSs or clear copulation attempts with the abdomen. From the total duration of courtship (*D*_C_) and aggression (*D*_A_) we calculated a behavioral contrast (*c*):

$$c=\frac{D_{\text{A}}-D_{\text{C}}}{D_{\text{A}}+D_{\text{C}}}$$

Positive *c* values indicate that the male spent more time with aggression, while negative values denote that the animal spent more time with courtship.

### Software Tools

All presented calculations were done in MATLAB R201 (The MathWorks Inc., Natick, MA, USA) running on a Java 1.6.0_17-b04 system (Sun Microsystems Inc.). The following toolboxes were used: MATLAB (Version 8.0), Simulink (Version 8.0), Box Counting (Version 1.10), Curve Fitting Toolbox (Version 3.3), Image Processing Toolbox (Version 8.1), Signal Processing Toolbox (Version 6.18), and Statistics Toolbox (Version 8.1). Video Annotation software was developed on Python 2.7.11^4^ employing the pygame site package.^5^

### Statistical Analysis

To test for significant differences between experimental groups, Fisher’s permutation test was applied to evaluate the differences of the medians of the respective measured variables. In some cases, we used a two-sided Kolmogorov–Simrnov test and once Fisher’s exact test (instances are indicated). *p*-Values were always corrected using the Benjamini–Hochberg false detection rate (71) procedure by applying the Matlab implementation of David M. Groppe.^6^

## Results

**Figure 2:**
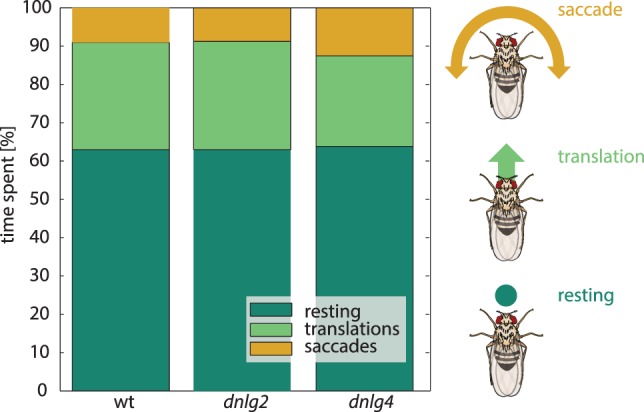
Locomotion. Distribution of the three fly-based velocity vectors representing a rotational movement, translation (forward movement), and resting, obtained by unsupervised k-means clustering [see Materials and Methods; (70)]. Colored boxes indicate the percentage of time spent with the respective behavior. Wild-type (wt) and Dnlg2-deficient flies spent equal times with the three behaviors. Dnlg4-deficient flies perform slightly less translation movements, though this effect is not significant [*N*(wt) = 97, *N*(dnlg2) = 96, *N*(dnlg4) = 66].

Heads of 50 Dnlg2-deficient and 50 Dnlg4-deficient flies were subjected to qPCR, revealing reduced levels of the respective *dlng* transcripts by 27 and 40% compared to wild type (see Table S1 in Supplementary Material), respectively. We subjected these flies to various tests to assess their behaviors. To identify general defects in mobility, we first monitored their spontaneous locomotion in a plain, circular arena. The locomotion trajectories were categorized using unsupervised k-means clustering as reported in Ref. (71, 72). The resulting movement categories were two “forward-sideways movements,” two “fast yaw turns,” and “resting” [see also Ref. (61)]. The distribution of these three categories showed no significant differences between wild type and the two mutant stains (Figure 2). Therefore, locomotion and its assembly from movement components seemed to be uncompromised by mutations in *dlng2* and *dlng4* and mutant defects in other behaviors are unlikely to result from general locomotion impairments.

**Figure 3:**
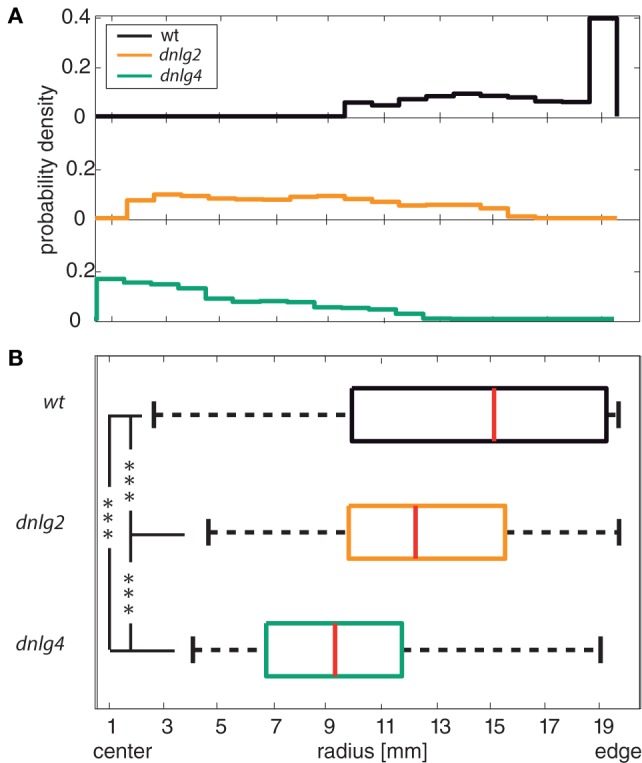
Center avoidance. **(A)** Histogram of the probability density within a circular arena for wild-type (*wt*), Dnlg2-deficient (*dnlg2^KO17^*), and Dnlg4-deficient (*dnlg4^LL01874/Def^*) flies. The plot indicates the probability density of the three fly lines in dependence to the position in the arena. 0 mm on the *y*-axis corresponds to the center of the arena; 20 mm corresponds to the edge of the arena. Wt shows a highly preferred presence at the edge of the arena and an avoidance of the center region. Dnlg2 and *dnlg4^LL01874/Def^* all display increased presence in central regions of the arena and a diminished probability density closer to the edge. **(B)** Boxplot of the median radius position of each individual. Red lines in box plots indicate the medians; boxes include 50% of the data set around the medians; whiskers include 1.5* interquartile distance and outliers are marked with red crosses (not shown). The median position is shifted significantly to the center of the arena in comparison to the wild type. We used a two-sample Kolmogorov–Smirnov test and corrected *p*-values *via* the Benjamini–Hochberg false FDR [*p*-values: *wt* vs *dnlg2^KO17^* 1.4845 × 10^−4^; *wt* vs dngl4 3.0372 × 10^−7^; *dnlg2^KO17^* vs dngl4 1.1854e × 10^−4^; *N*(*wt*) = 97, *N*(*dnlg2^KO17^*) = 96, *N*(*dnlg4^LL01874/Def^*) = 66].

The avoidance of central (open) areas of an arena is called centrophobia (73, 74). In *Drosophila*, impaired centrophobia has been associated with defects of the mushroom bodies (55). In contrast to wild-type flies that nearly exclusively circled around the edge of the arena, both *dnlg*-mutants often traversed the central part of the arena (Figure 3). Both mutant strains seemed to avoid the immediate vicinity of the walls, resulting in median radial positions that are significantly closer to the center than in *wt* (Figure 3B, two-sided Kolmogorov–Smirnov test *p* > 0.01). Dnlg4-deficient flies even displayed a weak tendency for preferred occupation of central regions.

**Figure 4:**
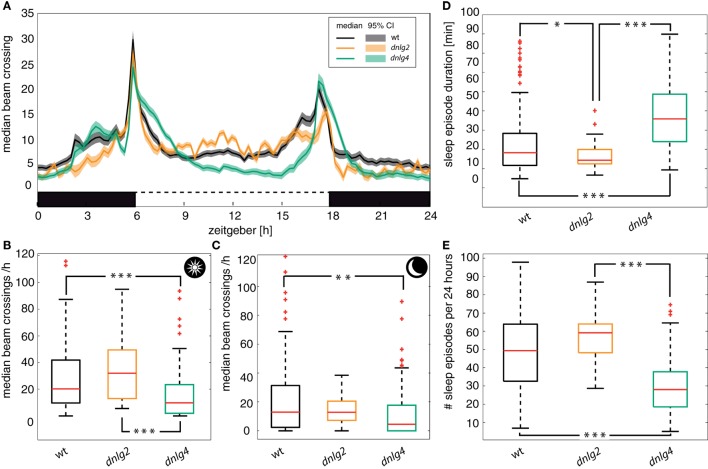
Circadian activity. **(A)** Average number of beam crossings of male flies during 5 days in a 12:12-h dark/light cycle. Boxes: black = dark, white = light. Solid lines: median; shaded areas: 95% confidence interval of the median. All genotypes show increased activity coinciding with dark/light and light/dark changes. The activity phases around the dark/light and light/dark changes are broadened or shifted in *dnlg4^LL01874/Def^*-mutant flies. **(B)** Diurnal activity based on the median number of beam crossings per hour. Panels **(B,C)** include data of the 10-h period around noon or midnight, respectively, excluding the hours with light changes and activity peaks. **(D)** Median duration of a sleep episode. A sleep episode was defined by the absence of beam crossings in a time window of 5 min. Dnlg2-deficient flies have significant lower sleep duration compared to *wt* (Kolmogorov–Smirnov Test corrected by Benjamini–Hochberg *p* = 0.0269) or Dnlg4-deficient flies (*p* = 9.54 × 10^−8^). The Dnlg-4-deficient flies sleep duration even surpasses that of the *wt* (*p* = 4.1 × 10^−13^). **(E)** Number of sleep episodes in a 24-h cycle. Dnlg4-deficient flies show less sleep episodes that the wild type (*wt* vs *dnlg4^LL01874/Def^ p* = 1.51 × 10^−12^; *dnlg2^KO17^* vs *dnlg4^LL01874/Def^ p* = 8.84 × 10^−7^). Dnlg2-deficient flies just miss significance at a *p*-value of 0.065.

A previous study demonstrated alterations of sleep in *dnlg4^LL01874/Def^*-mutant flies (54). We, thus, analyzed activity patterns and found Dnlg4-deficient flies have longer, but fewer episodes of sleep compared to wild-type and Dnlg2-deficient flies (see Figures 4D,E). Dnlg2-deficient flies, however, seemed to show an opposite phenotype with more but shorter sleep episodes than the wild type. Wild-type and both Dnlg-deficient fly strains displayed activity peaks associated with, or slightly preceding, the dark-to-light and light-to-dark switches (Figure 4A). In *dnlg4^LL01874/Def^*, these peaks lasted longer than in wild type and *dnlg2^KO17^*-mutants (Figure 4A); their overall activity was significantly reduced compared to both other strains during light periods (Figure 4B) and compared to wt during dark periods (Figure 4C). Compared to wt, Dnlg2-deficient flies displayed a slight but not significant increase of center crossing activity during light phases (Figure 4B) and no difference during dark periods (Figure 4C). In summary, overall activity is reduced in *dnlg4^LL01874/Def^* flies compared to wild-type flies (Fisher’s permutation test corrected with Benjamini–Hochberg fdr; *p*-value 0.00015) and *dnlg2* (*p* = 0.00008), while *dnlg2^KO17^* flies show a tendency for increased activity that fails significance (*p* = 0.09102) at least during periods of light.

**Figure 5:**
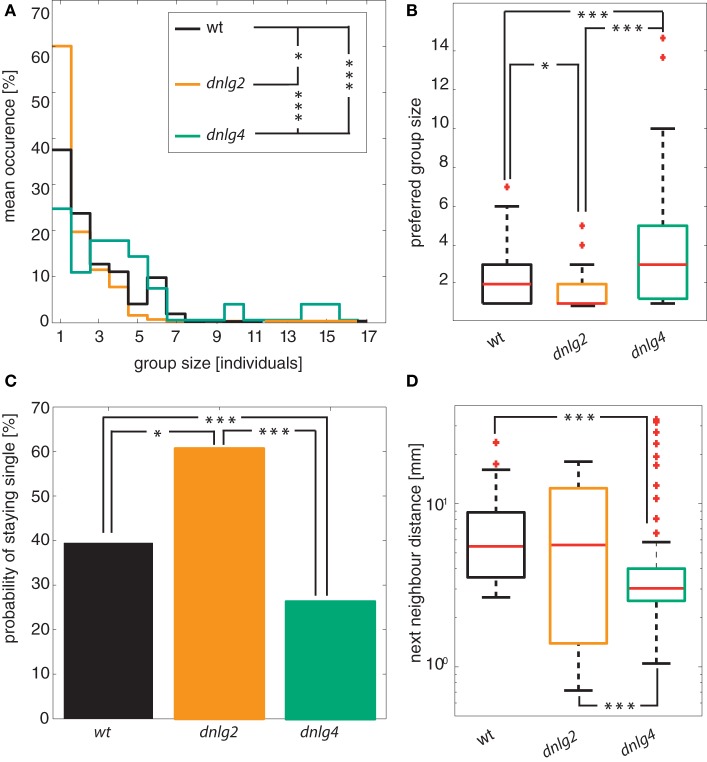
Group interactions. **(A)** Stair histogram of different group sizes. The histograms are significantly different [two-sided Kolmogorov–Smirnov test corrected by Benjamini–Hochberg fdr; *wt* vs *dnlg2^KO17^ p* = 0.0198; *wt* vs *dnlg4^LL01874/Def^ p* = 3.2577 × 10^−4^; *dnlg2^KO17^* vs *dnlg4^LL01874/Def^ p* = 2.5584 × 10^−9^; *N*(*wt*) = 104, *N*(*dnlg2^KO17^*) = 94, *N*(*dnlg4^LL01874/Def^*) = 119]. **(B)** Median of the preferred group size of each individual. While Dnlg4-deficient flies aggregate in significantly larger groups than *wt*, Dnlg2-deficient flies aggregate in significantly smaller groups [Kolmogorov–Smirnov test corrected by Benjamini–Hochberg fdr; *wt* vs *dnlg2^KO17^ p* = 3.26 × 10^−4^; *wt* vs *dnlg4^LL01874/Def^ p* = 0.0198; *dnlg2^KO17^* vs *dnlg4^LL01874/Def^ p* = 2.56 × 10^−9^; *N*(*wt*) = 104, *N*(*dnlg2^KO17^*) = 94, *N*(*dnlg4^LL01874/Def^*) = 119]. **(C)** Probability to stay alone during the group assay. Compared to wild-type (*wt*), Dnlg2-deficient flies (*dnlg2^KO17^*) have a significantly increased tendency to stay alone 60% (only 39% in *wt*) (Fisher’s exact test *p*-value 0.0065). In contrast, the chance to not associate in a group is at 24% for Dnlg4-deficient flies [Fisher’s exact test; *wt* vs *dnlg4^LL01874/Def^ p* = 0.0304; *dnlg2^KO17^* vs *dnlg4^LL01874/Def^ p* = 5.6103 × 10^−7^; *N*(*wt*) = 104, *N*(*dnlg2^KO17^*) = 94, *N*(*dnlg4^LL01874/Def^*) = 119]. **(D)** Average interindividual distance to the nearest neighbor within groups of animals. While *wt* and *dnlg2^KO17^* flies maintain similar interindividual distances, *dnlg4^LL01874/Def^* flies assume significantly closer positions with respect to *wt* and *dnlg2^KO17^*.

Previously, Dnlg2-deficient flies reportedly displayed abnormal social and mating behavior (16). In order to study altered attraction to conspecifics, we recorded the location data of 24 animals (per replicate) that were allowed to successively enter a circular arena from two opposite sides in 90-s intervals. To assess group formation, we used their positional data and subjected it to a hierarchical agglomerative clustering. While wild-type males remained single with a probability of 39%, this probability was significantly increased in Dnlg2-deficient flies (60%) and reduced to 24% in Dnlg4-deficient flies [Figure 5C; Fisher’s exact test *p*-value *wt* vs *dnlg2^KO17^* = 0.0065; *wt* vs *dnlg4^LL01874/Def^p* = 0.0304; *dnlg2^KO17^* vs *dnlg4^LL01874/Def^p* = 5.6103 × 10^−7^; *N*(*wt*) = 104, *N*(*dnlg2^KO17^*) = 94, *N*(*dnlg4^LL01874/Def^*) = 119]. Wild-type flies and *dnlg2^KO17^*-mutants associated in groups of up to seven individuals whereas group size shifted to larger values in *dnlg4^LL01874/Def^*-mutants with up to 15 animals per group [Figure 5A; differences in group sizes tested with a two-sided Kolmogorov–Smirnov test (*wt* vs *dnlg2^KO17^p* = 0.0198; *wt* vs *dnlg4^LL01874/Def^p* = 3.2577 × 10^−4^; *dnlg2^KO17^* vs *dnlg4^LL01874/Def^p* = 2.5584 × 10^−9^; *N*(*wt*) = 104, *N*(*dnlg2^KO17^*) = 94, *N*(*dnlg4^LL01874/Def^*) = 119; *p*-values corrected after (70))]. Chaining behavior was excluded from this analysis, because this phenomenon was caused by an improper termination of courtship and not a direct effect on aggregation behavior. We also calculated the average distances of animals to their nearest neighbor within such groups. Compared to wild-type and *dnlg2^KO17^*-mutant males that maintained similar interindividual distances, the distance was reduced in Dnlg4-deficient flies (Figure 5D). Hence, although interindividual distances are only changed in *dnlg4^LL01874/Def^*-mutants, Dnlg2-deficient males have a lower tendency to form groups, while *dnlg4^LL01874/Def^*-mutants show an increased tendency to aggregate (Figure 5B).

**Figure 6:**
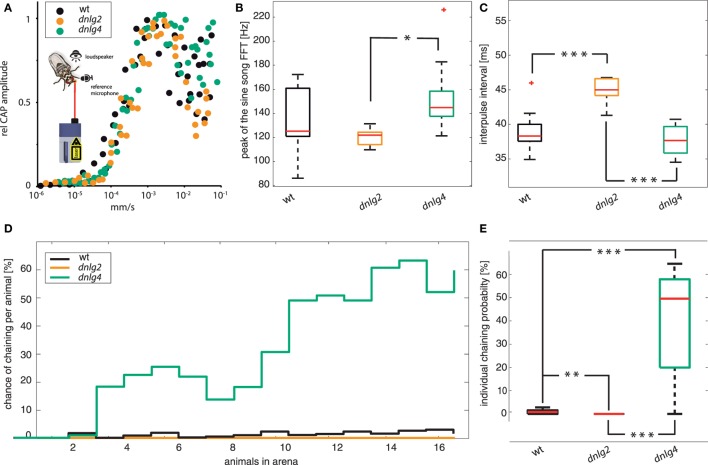
Auditory function, courtship songs, and chaining. **(A)** Relative compound action potential (CAP) amplitude of tone-evoked auditory nerve responses plotted against the intensity of the tone. Tone frequencies were matched to the individual best frequencies of the antennal sound receiver. No distinct shift in the response threshold could be detected when comparing wild-type (*N* = 5), Dnlg4-deficient (*N* = 5), and Dnl2g-deficient (*N* = 3) flies. The insect shows a general schematic with which the antennal movements and sounds were recorded as references during the electrophysiological recordings. **(B)** Dominant frequency component of sine songs. Significance was calculated using Fisher’s permutation test for differences of medians, corrected with Benjamini–Hochberg false detection rate procedure [*dnlg2^KO17^* vs *dnlg4^LL01874/Def^ p*-value = 0.0195; *N*(*wt*) = 17; *N*(*dnlg2^KO17^*) = 10; *N*(*dnlg4^LL01874/Def^*) = 17]. **(C)** Period durations (=inter-pulse intervals) of pulse songs [identical test for significance; *wt* vs *dnlg2^KO17^ p* = 2 × 10^−4^; *dnlg2^KO17^* vs *dnlg4^LL01874/Def^ p* = 8 × 10^−5^; *N*(*wt*) = 16, *N*(*dnlg2^KO17^*) = 10, *N*(*dnlg4^LL01874/Def^*) = 17] **(D)** Probability of male–male chaining (a chain consists of two or more flies that chase each other with one extended wing) behavior per animal in dependence to the number of animals being present in the arena. In *wt*, the chance for male–male chaining behavior increases slightly with increasing numbers of present animals but never exceeds 4%. While *dnlg2^KO17^*-mutants never engaged in courtship chains, *dnlg4^LL01874/Def^*-mutants showed increased chaining behavior, reaching maximum probabilities of over 60% [*N*(*wt*) = 104, *N*(*dnlg2^KO17^*) = 94, *N*(*dnlg4^LL01874/Def^*) = 119]. **(E)** Boxplots depicting the individual probability to be part of male courtship chain. Dnlg4 shows a significantly increased chance, while *dnlg4^LL01874/Def^* shows a significant decrease [Kolmogorov–Smirnov test; *wt* vs *dnlg2^KO17^ p* = 7.3279 × 10^−5^; *wt* vs *dnlg4^LL01874/Def^ p* = 6.0598 × 10^−9^; *dnlg2^KO17^* vs *dnlg4^LL01874/Def^ p* = 1.7207 × 10^−9^; *N*(*wt*) = 104, *N*(*dnlg2^KO17^*) = 94, *N*(*dnlg4^LL01874/Def^*) = 119].

Courting *D. melanogaster* produce two types of songs, a sine song and a pulse song. These acoustic communication signals play important roles in driving female mating decisions. By comparing songs between the three strains, we found that mutations in both *dnlg2^KO17^* and *dnlg4^LL01874/Def^* affect the songs. The major frequency component of sine songs was quite variable in wild type, ranging between 120 and 160 Hz (Figure 6B). While the dominant sine song frequency was slightly lowered in Dnlg2-deficient flies, Dnlg4-deficient flies produced songs with higher sine song frequencies (*dnlg2^KO17^* vs *dnlg4^LL01874/Def^p* = 0.00625; Fisher’s permutation test, corrected by Benjamini–Hochberg fdr). Analysis of courtship pulse songs revealed no differences in amplitude and shape (number of oscillations per pulse) between wild-type males and the two *dnlg*-mutants, suggesting that the neuromuscular components that generate acoustic communication signals were not compromised by the mutations in Dnlgs. Interpulse intervals had median durations of 38 ms in wild types and 37 ms in *dnlg4^LL01874/Def^* but were significantly longer in CSs of *dnlg2^KO17^*-mutants (Figure 6C).

We, next, analyzed male-chaining behavior, whereby males follow each other with one extended wing (75). The probability of wild-type males to engage in male-directed courtship was generally low and remained below 5% even when the arena was filled with larger numbers of individuals (Figures 6D,E). Chaining, however, was entirely absent in Dnlg2-deficient flies and thereby significantly lower compared to wt (*p*-value > 0.001; Kolmogorv–Smirnov). In contrast, *dnlg4*-mutants formed courtship chains that included up to 17 animals, and the probability of individual flies to engage in chaining increased significantly (*p*-value > 0.001; Kolmogorv–Smirnov), reaching more than 60% as the number of animals in the arena was increasing (Figure 6D). In summary, absence of Dnlg2 and Dnlg4 not only affects male CSs, but also chain formation, a male-directed courtship behavior.

**Figure 7:**
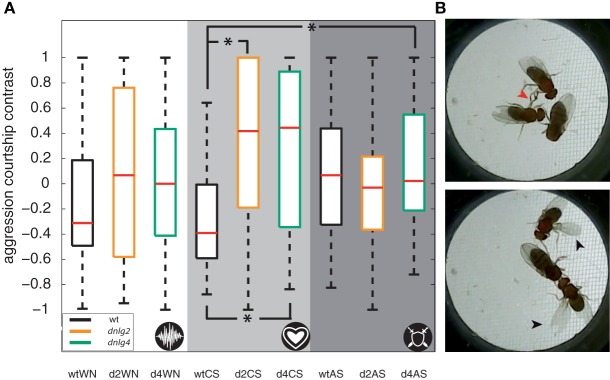
Competitive courtship assay with acoustic stimulation. **(A)** Boxplots of the aggression courtship contrast (for definition, see Materials and Methods). The flies were stimulated by either white noise (white backdrop), courtship songs (CSs) (light gray backdrop), or aggression sound (dark gray background), as also indicated by the icon in lower right corner. Dnlg2- and Dnlg4-deficient flies reacted with significantly higher amounts of aggression behavior when stimulated with CSs than *wt* [Fisher’s permutation test for differences of medians, corrected with Benjamini–Hochberg fdr; *wt* vs *dnlg2^KO17^ p* = 0.024; *wt* vs *dnlg4^LL01874/Def^ p* = 0.027; *N*(*wt*) = 20; *N*(*dnlg4^LL01874/Def^*) = 20; *N*(*dnlg2^KO17^*) = 20] **(B)** Two example frames that were manually assessed. The red arrow in the upper frame indicates leg fencing, a typical male–male aggression behavior. The black arrows in the lower frame indicate unilateral wing extension, a hallmark of courtship behavior.

Hahn et al. (16) reported reduced social interactions in Dnlg2-deficient flies in competitive courtship assays where two males switched between male-directed agonistic and female-directed behavior. A recent study (76) further reported that AS promote aggression, while CSs inhibit aggressive interactions between *Drosophila* males. In order to test whether Dnlg2- and Dnlg4-deficient flies react appropriately to sound signals, we extended the competitive courtship paradigm by continuous stimulation with WN, CS, or aggression sounds. Prior to the experiments, we assayed hearing organ function in the flies, revealing that auditory mechanics and sound-induced auditory nerve response in both mutant strains resemble those of wild-type flies (Figure 6A). During stimulation with WN and CSs *wt* males spent more time courting the female than displaying aggression against the other male (see Figure 7). During stimulation with aggression sounds, the latter male-directed aggression was increased [Figure 7A; Fisher’s exact test corrected with Benjamini–Hochberg fdr; *p*-value *wt*(white noise) vs *wt*(aggression sounds) = 0.0237]. Neither Dnlg2- or Dnlg4-deficient males altered the frequencies of aggressive and courtship behavior during stimulation with aggressive sounds. Stimulation with CSs slightly increased wt courtship behavior, whereas the opposite effect was seen in Dnlg2- and Dnlg4-deficient males, which reduced their courtship significantly and increased aggression (*wt* vs *dnlg2^KO17^p* = 0.0237; *wt* vs *dnlg4^LL01874/Def^p* = 0.0237). Hence, both Dnlg2- and Dnlg4-deficient flies seem to fail to respond appropriately to sounds.

## Discussion

The presented behavioral data suggest that the trans-synaptic adhesion molecules Dnlg2 and Dnlg4 may play a prominent role in the neuronal regulation of *Drosophila*’s social interactions. *dnlg2* and *dnlg4* are both expressed at central nervous synapses, but Dnlg2 is also present at neuromuscular synapses (53). Of the other two *Drosophila* Nlgs, *dnlg1* is expressed at neuromuscular postsynapses (77) and *dnlg3* is expressed in neuromuscular junctions and the central nervous system (78). Similar to Nlgs in mammalian central nervous systems, Dnlg1, Dnlg2, and Dnlg3 seem important for synaptic maturation and functional maintenance (studied at the neuromuscular junction) rather than being crucial for synaptogenesis.

In order to assess the requirements of Nlgs for the proper functionality of neural circuits, we analyzed the effects of mutations in *dlng2* and *dlng4* on walking, hearing, sound production, and social behavior. Electrophysiological recordings from antennal auditory nerves detected no differences in auditory sensitivity between wild type and mutant flies. Intact chemosensation can also be assumed, since males of both mutants correctly addressed females with courtship and males with agonistic behaviors in competitive courtship assays. In *Drosophila*, sex recognition and the assessment of female reproductive state has been demonstrated to largely depend on the detection of sex- and state-specific surface hydrocarbons (79, 80). In addition, *dnlg2^KO17^*- and *dnlg4^LL01874/Def^*-mutants, like wild-type flies, maintained peaks of locomotor activity when lights were switched on or off [(16, 54), this study]. Nonetheless, the sleep rhythms of Dnlg2- and Dnlg4-deficient flies were altered in an opposing manner, with Dnlg4-deficient flies sleeping more often and shorter than *wt*, consistent with previous studies (54), and Dnlg2-deficient flies sleeping less often with longer duration (Figure 4). Notwithstanding seemingly normal sensory functions, both *dnlg2^KO17^*- and *dnlg4^LL01874/Def^*-mutants thus show opposing deficits in sleep.

Unlike other insect species, such as locusts and cockroaches, that contain both excitatory glutamatergic and inhibitory GABAergic motor neurons, *Drosophila* only possesses excitatory neuromuscular synapses (81). Synaptic expression of *dnlgs* 1, 2, and 3 and consequences for synaptic transmission resulting from the lack of these Nlgs at the *Drosophila* neuromuscular junction have been described in previous studies (53, 77, 78, 82). The *dnlg2^KO17^*-mutants used in our experiments displayed no defects that could be attributed to compromised neuromuscular transmission. Their walking behavior included all typical movement components (saccades, straight translation, and intermittent rest) that were combined in a coordinated fashion resembling that of wild-type flies (61). Moreover, CS pulses contained similar numbers of oscillations of the extended wing in *dnlg2^KO17^*-mutants and wild-type flies, indicating normal neuromuscular function. Altered repetition rates of courtship pulses and altered sine song frequencies in *dnlg2^KO17^* point to alterations of central nervous circuits that determine the rhythm of wing movements. Along this line, Clyne and Miesenböck (83) demonstrated that initiation of sine and pulse song is triggered by descending brain neurons, whose activity synchronizes the intrinsic activity of thoracic pattern generators to a faster central clock. Avoidance of open areas is regarded as a measure for anxiety in various animal models (84–87). Wild-type *Drosophila* exhibit an obvious centrophobism that critically depends on the functionality of the mushroom bodies (55). While total ablation of mushroom bodies reduced centrophobism, specific inactivation of mushroom body γ-lobes increased centrophobistic behavior (55). Both, *dnlg*-mutant strains used in this study showed reduced centrophobic behavior (Figure 3) and *dnlg2* is expressed in mushroom bodies along with *dlng4* (unpublished immunocytochemical data by W. Xie), in the central complex (54). Both mushroom bodies and central complex are involved in the regulation of higher order social behaviors (55–59), so expression patterns seem consistent with the observed behavioral defects.

During an unsupervised and data-derived group formation analysis, 20 mm emerged as the distance threshold for group interactions. This is nearly identical to the distance at which a conspecific fly is only detected by a single ommatidium in the complex eye of *Drosophila* (61, 88, 89). Even though courtship and potentially other social contexts depend also on other sensory modalities [e.g., olfaction (90)], vision seems to be the most accurate and direct sense to judge the interindividual distance. Therefore, it is not surprising that group formation might be limited by the visual acuity of *Drosophila*. Aggregation of individuals and the formation of groups is a basic feature of social interaction, as for example oviposition in female *Drosophila* is dependent on group size (91). Compared to wild-type males, *dnlg2^KO17^*-mutants displayed a reduced tendency to form groups but those who accumulated in groups assumed similar minimal interindividual distances (Figure 5). In contrast, *dnlg4^LL01874/Def^*-mutants had a lower tendency to remain single, formed larger groups, and assumed closer positions to other group members.

Furthermore, this closer interindividual distance might have led to an increased formation of courtship chains in Dnlg4-deficient flies (Figure 6). Dnlg2-deficient flies showed a significantly lower chance of male–male courtship, which might be caused by their larger interindividual distance (Figure 6). It has been shown that CSs stimulate the formation of courtship chains (92, 93) and especially the inter-pulse interval of CSs has been identified as a critical factor for species recognition and attractiveness (20, 94), an alteration in song production might also lead to the chaining phenotype. Since *dnlg2^KO17^*-mutant males produced songs with significantly prolonged inter-pulse intervals (Figure 6C), complete absence of chaining behavior could also have been a consequence of less attractive songs. Thus, the chaining phenotypes might be epiphenomena of the altered interindividual distance and CS production.

Absence of Dnlg2 and Dnlg4 altered social interactions of *Drosophila* males, without causing obvious impairments of sensory functions and execution of movements [this study, (16)]. Deficiency of Dnlg2 and Dnlg4, which seem to be differentially expressed at excitatory and inhibitory synapses, induced opposing deviations from wild-type behaviors in some behavioral paradigms, such as sleep rhythm, male chaining, group size, and interindividual distance. Other behavioral paradigms, such as center avoidance and stimulation with *wt* CSs, revealed equally altered behavior in a non-opposing fashion. Thus, Dnlg2 and Dnlg4 may play different roles in the regulation of synaptic transmission within brain neuropils implicated in the social behavior of *D. melanogaster*.

Like fly Nlgs, mammalian ones are differentially expressed at different types of synapses, and deletion or overexpression of particular Nlgs resulted in altered proportions of excitatory vs inhibitory transmission in brain neuropils (28, 51). Both overrepresentation of excitation and overrepresentation of inhibition have been associated with ASD phenotypes (48) and have also been observed in mouse models of ASD (95–97). Targeted manipulation of *Drosophila* Dnlg2 and Dnlg4 functions in specific brain regions might help to identify the neural circuits that regulate social behaviors and to assess the role of Nlgs in the balance between neuronal excitation and inhibition.

## Author Contributions

All authors designed the study. KC, AH, RK, IG, NH, RH, and BG collected and analyzed the data, HG and BG designed the behavioral setups and data acquisition. RH and BG wrote the manuscript, and all authors edited and approved the manuscript. KC and AH contributed equally.

## Conflict of Interest Statement

The authors declare that the research was conducted in the absence of any commercial or financial relationships that could be construed as a potential conflict of interest.

## Footnotes

^1^https://de.mathworks.com/matlabcentral/fileexchange/31274-fast-k-means.

^2^http://trikinetics.com.

^3^http://audacity.sourceforge.net.

^4^www.python.org.

^5^www.pygame.org.

^6^https://de.mathworks.com/matlabcentral/fileexchange/27418-fdr-bh.
