# Supplementary material for: Neuroligins Nlg2 and Nlg4 Affect Social Behavior in Drosophila melanogaster
Source: Front Psychiatry. 2017 Jul 10;8:113. doi: 10.3389/fpsyt.2017.00113 (PMC5502276; doi:10.3389/fpsyt.2017.00113)
Supplement: Supplementary file 1 [file Table_1.docx]

Supplementary Table 1: Expression levels of *dnlg2 and dnlg4* determined by qPCR. Expression levels are normalised to *Rpl32* reference gene expression. w/o RT: without reverse transcriptase.

|  | dnlg2^KO17^ primer | | | | dnlg4^LL01874/Def^ primer | | | |
| --- | --- | --- | --- | --- | --- | --- | --- | --- |
|  | avr. ct | Δ ct | ΔΔ ct | 2 ^-ΔΔ ct^ | avr. ct | Δ ct | ΔΔ ct | 2 ^-ΔΔ ct^ |
| CantonS | 25.82 | 8.17 | 0 | **100%** | 26.89 | 9.33 | 0.00 | **100%** |
| CantonS w/o RT | 40 | 0 | -9.3 | **0%** | 40 | 0 | -9.3 | **0%** |
| w^1118^ | 26.6 | 8.24 | 0.07 | **95.3%** | 27.82 | 9.53 | 0.2 | **87.3%** |
| w^1118^ w/o RT | 40 | 0 | -9.3 | **0%** | 40 | 0 | -9.3 | **0%** |
| dnlg | 25.25 | 10.07 | 1.9 | **26.9%** | 29.2 | 10.68 | 1.35 | **39.3%** |
| dnlg w/o RT | 40 | 0 | -9.3 | **0%** | 40 | 0 | -9.3 | **0%** |
| H_2_O | 40 | 0 | -9.3 | **0%** | 40 | 0 | -9.3 | **0%** |
