# Supplementary material for: Neuroligins Nlg2 and Nlg4 Affect Social Behavior in Drosophila melanogaster
Source: Front Psychiatry. 2017 Jul 10;8:113. doi: 10.3389/fpsyt.2017.00113 (PMC5502276; doi:10.3389/fpsyt.2017.00113)
Supplement: Supplementary file 2 [file Table_2.docx]

Supplementary Table 2: Overview of the primers used in the qRT PCR analysis.

| Symbol | Gene | Amplicon size [bp] | Ordering # / Sequence |
| --- | --- | --- | --- |
| RpL32 | Ribosomal protein L32 | 115 | QT00985677 |
| dnlg2^KO17^ | *Drosophila* neuroligin 2 | 147 | 5‘-GACGAGGACCAACAGCACGAAAG-3‘  5` GCATCGCCGTCATGGTTGGAC 3` |
| dnlg4^LL01874/Def^ | *Drosophila* neuroligin 4 | 190 | 5´ GCAGCAGCAGCAAGTGCCGCA 3´ 5´- ATCTGCATCCACTGGTGTCCCATTGA 3´ |
