# Supplementary material for: Neuroligins Nlg2 and Nlg4 Affect Social Behavior in Drosophila melanogaster
Source: Front Psychiatry. 2017 Jul 10;8:113. doi: 10.3389/fpsyt.2017.00113 (PMC5502276; doi:10.3389/fpsyt.2017.00113)
Supplement: Supplementary file 3 [file Image_1.pdf]

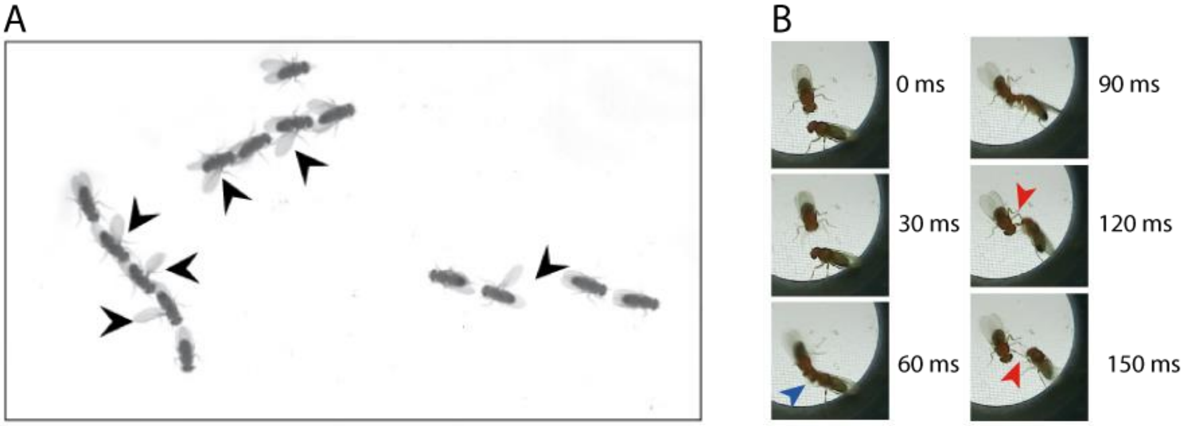

Supplementary Figure 1: Examples of manual video annotation A) Chaining dnlg4LL01874/del mutant males. Black arrows indicate wing extension, in this case directed towards another male. B) Excerpt form a recording, showing a male (upper left at time zero) pouncing (blue arrow) and attacking another male. After 90ms, the leg fencing (red arrows) is observed.
